# Identification of *Dw1*, a Regulator of Sorghum Stem Internode Length

**DOI:** 10.1371/journal.pone.0151271

**Published:** 2016-03-10

**Authors:** Josie Hilley, Sandra Truong, Sara Olson, Daryl Morishige, John Mullet

**Affiliations:** Department of Biochemistry and Biophysics, Texas A&M University, College Station, Texas, United States of America; Louisiana State University Agricultural Center, UNITED STATES

## Abstract

Sorghum is an important C4 grain and grass crop used for food, feed, forage, sugar, and biofuels. In its native Africa, sorghum landraces often grow to approximately 3–4 meters in height. Following introduction into the U.S., shorter, early flowering varieties were identified and used for production of grain. Quinby and Karper identified allelic variation at four loci designated *Dw1*-*Dw4* that regulated plant height by altering the length of stem internodes. The current study used a map-based cloning strategy to identify the gene corresponding to *Dw1*. Hegari (*Dw1dw2Dw3dw4*) and 80M (*dw1dw2Dw3dw4*) were crossed and F_2_ and HIF derived populations used for QTL mapping. Genetic analysis identified four QTL for internode length in this population, *Dw1* on SBI-09, *Dw2* on SBI-06, and QTL located on SBI-01 and SBI-07. The QTL on SBI-07 was ~3 Mbp upstream of *Dw3* and interacted with *Dw1*. *Dw1* was also found to contribute to the variation in stem weight in the population. *Dw1* was fine mapped to an interval of ~33 kbp using HIFs segregating only for *Dw1*. A polymorphism in an exon of Sobic.009G229800 created a stop codon that truncated the encoded protein in 80M (*dw1*). This polymorphism was not present in Hegari (*Dw1*) and no other polymorphisms in the delimited *Dw1* locus altered coding regions. The recessive *dw1* allele found in 80M was traced to Dwarf Yellow Milo, the progenitor of grain sorghum genotypes identified as *dw1*. *Dw1* encodes a putative membrane protein of unknown function that is highly conserved in plants.

## Introduction

Sorghum is the fifth most widely cultivated cereal crop worldwide. This C4 grass is grown for grain, feed, forage, sugar, and biofuels. Sorghum diverged from a common ancestor with maize ~12 MYA and rice ~50 MYA [1]. It is native to Africa and parts of India and Australia with most African landraces growing to 3–4 meters in height before harvest. When grown in the U.S., many sorghum accessions from Africa produce tall, late flowering plants. However, after its initial introduction to the U.S., breeders found naturally occurring shorter genotypes that were subsequently used to breed short grain sorghum varieties to reduce stalk lodging. Sorghum genotypes with longer stems are grown for forage, sugar, and biomass to increase yield. Energy sorghum hybrids are 3–4 meters in height with long internodes and biomass yield ranging from 15–40 Mg/ha depending on genotype and environment [2–4]. Stem biomass of a first generation energy sorghum hybrid accounted for ~80% of harvested shoot biomass [5]. Therefore, a more complete understanding of the genetic and biochemical basis of stem growth could identify ways to increase the stem biomass yield of bioenergy sorghum.

Plant height is affected by the length of each internode, the rate of internode production, and the duration of vegetative growth. The latter influences height because production of internodes stops at floral induction even though internode elongation continues until anthesis. In the 1950s, Quinby and Karper [6] identified four loci, *Dw1-Dw4*, that control height by modifying internode length. Recessive alleles at the four loci reduce internode length [6]. Pleiotropic effects of *Dw2* and *Dw3* have been reported and include panicle length, seed weight, and leaf area for the former [7,8] and seed weight, panicle size, tiller number, and leaf angle for the latter [8–10]. However, pleiotropic effects have not been described for *Dw1* or *Dw4*. Additionally, QTL for height, including *Dw3* and a QTL on chromosome 9, have been found to co-localize with QTL for stem and total biomass [11].

The gene corresponding to *Dw3* was cloned by Multani et al. [12] and determined to encode an ABCB1 auxin efflux transporter. Further analysis showed that the maize homolog, *br2*, transports auxin from intercalary meristems located at the base of a stem internode into the elongating internode [13]. QTL corresponding to *Dw1* and *Dw2* have been identified, but the underlying genes are unknown. *Dw1* was mapped to the distal end of SBI-09 [14] and *Dw2* to SBI-06 adjacent to *Ma1* [15]. Recently, a QTL for stem length was identified on SBI-07 located near *Dw3* in a RIL population from a cross of Tx430 and P898012 [16].

The Green Revolution dwarfing genes in rice and wheat reduce gibberellin induced stem elongation producing semi-dwarf varieties with reduced lodging. In rice, semi-dwarf genotypes were found to encode a less active version of gibberellin 20 oxidase, an enzyme involved in GA synthesis [17]. In wheat, dwarf varieties contain alleles of a gene encoding a DELLA protein that is involved in gibberellin (GA) signaling [18]. Because of this, several researchers have suggested that *Dw1* encodes a gibberellin 2 oxidase that is located in the genomic region near SNPs associated with this height locus on SBI-09 [19–21]. However, recent work showed that gibberellin mutants in sorghum have bent stems, which are not observed in genotypes recessive for the sorghum dwarfing genes. Furthermore, there were no sequence variants in the GA2 oxidase coding region located on SBI-09 near *Dw1* between genotypes that were *Dw1* and *dw1* [22].

In this study, the gene corresponding to *Dw1* was map-based cloned using an F_2_ population and HIFs derived from Hegari and 80M. *Dw1* encodes a protein of unknown function that is highly conserved in plants. In the process of identifying *Dw1*, a QTL that modulates internode length was identified on SBI-01 and a QTL on SBI-07 corresponding to one recently identified by Li et al. [16] was found to interact with *Dw1*.

## Methods

### QTL Mapping of Stem Traits in Hegari x 80M

A map-based cloning approach was used to identify the gene corresponding to *Dw1*. A population segregating for *Dw1* was constructed by crossing Hegari (*Dw1dw2Dw3dw4*) and 80M (*dw1dw2Dw3dw4*) [23]. The F_1_ plants were selfed and the F_2_ population (n = 218) was planted in April 2011 and grown in a greenhouse in long days (14 hours light, 10 hours dark), three plants per 3.8 gallon pot in soil that was a mixture of vermiculite (Sun Gro Horticulture) and Belk Clay soil (2:1) obtained from the Texas A&M University Field Station west of College Station, Texas. Osmocote Classic 13-13-13 (Scotts) was mixed into the soil and plants were subsequently fertilized every two weeks with Peters General Purpose 20-20-20 (JR Peters, Inc.). Plants were phenotyped for days to flowering, total stem fresh and dry weight, total stem length, and length and diameter of each internode at grain maturity for early flowering plants and after 190 days of growth for late flowering genotypes. DNA was extracted from leaf tissue using the FastDNA Spin Kit (MP Biomedicals). Each plant was genotyped using Digital Genotyping [24], using the enzyme FseI for digesting the genomic DNA. The Illumina GAII was used for sequencing and the reads were mapped onto the *Sorghum bicolor* genome v1.0 (Phytozome v6).

A genetic map for this population was constructed using MapMaker [25], with the Kosambi function. QTL analysis was performed in QTL Cartographer [26] using Composite Interval Mapping with a walk speed of 1.0cM and forward and backward model selection. The threshold was set using 1000 permutations and α = 0.05. QTL mapping was performed with the entire population, early flowering plants only (n = 85), and late flowering plants only (n = 118). To look for possible gene interactions multiple-QTL analysis was used. A single QTL analysis using the EM algorithm initially identified four primary additive QTL which were used to seed model selection. The method of Manichaikul et al. [27] was employed for model selection as implemented in R/qtl [28] for multiple-QTL analysis. Computational resources on the WSGI cluster at Texas A&M were used to calculate the penalties for main effects, heavy interactions, and light interactions. These penalties were calculated from 24,000 permutations for the average internode length to find a significance level of 5% in the context of a two-dimensional, two-genome scan.

### Fine Mapping of *Dw1*

To refine the location of *Dw1*, plants were selected from early flowering lines that were segregating for *Dw1*, but fixed for the other loci controlling internode length. These plants (n = 6) were selfed to create Heterogeneous Inbred Families (HIFs) [29]. For each family, the F_3_ plants (n = 75 for each HIF) were planted in December 2011 and grown in the greenhouse as with the F_2_ population, phenotyped as described above, and genotyped using Digital Genotyping. The phenotypes were used to classify plants as dominant, heterozygous, or recessive at *Dw1*. The phenotype data were then correlated with genotype data spanning *Dw1*. The region encoding *Dw1* was further refined using F_4_ HIFs derived from F_3_ plants that were heterozygous at *Dw1*. The plants were planted in June 2013 and grown in the greenhouse as with the previous generations, except in Sunshine MVP soil (Sun Gro Horticulture). At grain maturity the plants were phenotyped for stem and internode length (n = 78 for each HIF). The population was screened for individuals with breakpoints in the delimited *Dw1* region using two CAPS (Cleaved Amplified Polymorphic Sequence) markers, except for Family 2 which was genotyped using Digital Genotyping because one of the CAPS markers was fixed in that family. The CAPS markers are described in S1 Table. Restriction enzyme digests were performed using the manufacturer’s recommended temperature for each enzyme (New England Biolabs) and incubations of at least 2 hours. All PCR amplification was done with Phusion (New England Biolabs). The breakpoints were refined using SNPs that were genotyped through Sanger sequencing using Big-Dye Terminator cycle sequencing kit v3.1 (Invitrogen) (S1 Table).

### Sequencing of Candidate Genes

All of the genes in the region encoding *Dw1* delimited by fine mapping were sequenced in the parental genotypes used for *Dw1* mapping as well as Standard Yellow Milo (*Dw1Dw2Dw3dw4*) and Dwarf Yellow Milo (*dw1Dw2Dw3dw4*) by Sanger sequencing. The yellow milos are nearly isogenic except at *Dw1*. The primers used to amplify and sequence genes in the delimited *Dw1* region are listed in S2 Table. A polymorphism in Sobic.009G229800 that distinguished 80M and Hegari created a stop codon and truncated protein in 80M (*dw1*).

### cDNA Sequencing and qRT-PCR

RNA was collected from stem tissue for cDNA sequencing and to characterize the expression of *Dw1* (Sobic.009G229800). The two parents (n = 3 for each) were planted in the greenhouse in August 2013, and after 42 days of growth, stem tissue was collected from plants in the mid-morning. Plants were cut at soil level and leaves and leaf sheaths were quickly stripped from the stem. Internodes that were in the process of elongating were located and divided into an upper portion of the internode that had stopped elongating, a mid-lower region containing cells that are in the process of elongation, and the base of the internode containing the intercalary meristem. A fully expanded internode was also harvested. The tissue was ground in liquid nitrogen and the RNA extracted using a Direct-zol RNA kit (Zymo Research) with TRI-Reagent (Molecular Research Center). The RNA was quantified on the Nanodrop spectrophotometer. RNA quality was confirmed by visualizing final samples with the BioAnalyzer (Agilent Technologies). Two technical replicates of cDNA and a no reverse transcriptase control were made using SuperScript III primed with both random hexamers and oligo (dT) at a ratio of 9:1 from 1μg of RNA.

Sobic.009G229800 cDNA from elongating stem tissue from each parental genotype was Sanger sequenced. The primers used to sequence the cDNA are listed in S3 Table. Gene expression was analyzed using qRT-PCR on the 7900HT Fast Real-Time PCR System (Applied Biosystems) running SDS v2.3 software. *Dw1* was amplified in the presence of SYBR green using the following conditions: hold at 95°C for 10 mins, 40 cycles of 95°C 15 sec. and 60°C for 1 min. Primer efficiencies were determined based on a standard curve from a serial dilution of five 10-fold dilutions of PCR product for each parent. Primer specificity was checked using a dissociation curve and running PCR products on a gel. The primers used for *Dw1* amplification were: 5’-TACGCTAAAGATGGCACAAGTC-3’ and 5’-TCCTTTGAACACGTCCAAGC-3’. The data was analyzed according to the comparative Ct (ΔΔCt) method [30] using the 18S ribosomal RNA to normalize the expression values and the sample from the 80M mature tissue as the calibrator. 18S ribosomal RNA reactions were performed with the TaqMan rRNA primers and probe (Applied Biosystems) and TaqMan MasterMix. Three technical replicates of qPCR were performed for each sample. The three biological replicates were averaged and the standard error of the mean calculated.

### Protein Sequence Analysis

To gain insight into the function of *Dw1*, the protein sequence translated from the Hegari cDNA sequence was compared to other plants, using BLAST in Phytozome v.10 and to the NCBI database using NCBI BLAST. A sequence comparison of the protein’s homologs in maize, rice, and Arabidopsis was generated in Jalview [31] using T-Coffee [32] with default settings. A phylogenetic tree of several protein homologs was constructed with MEGA6 [33] using MUSCLE [34,35] to align the sequences and Maximum Likelihood to construct the tree. Protein function and structure was examined using several web-based programs: PSIPRED-MEMSAT-SVM [36,37], PSIPRED-DISOPRED[38], PONDR [39], and FoldIndex [40] using default settings for each program.

## Results

### QTL Mapping of Stem Traits

**Fig 1:**
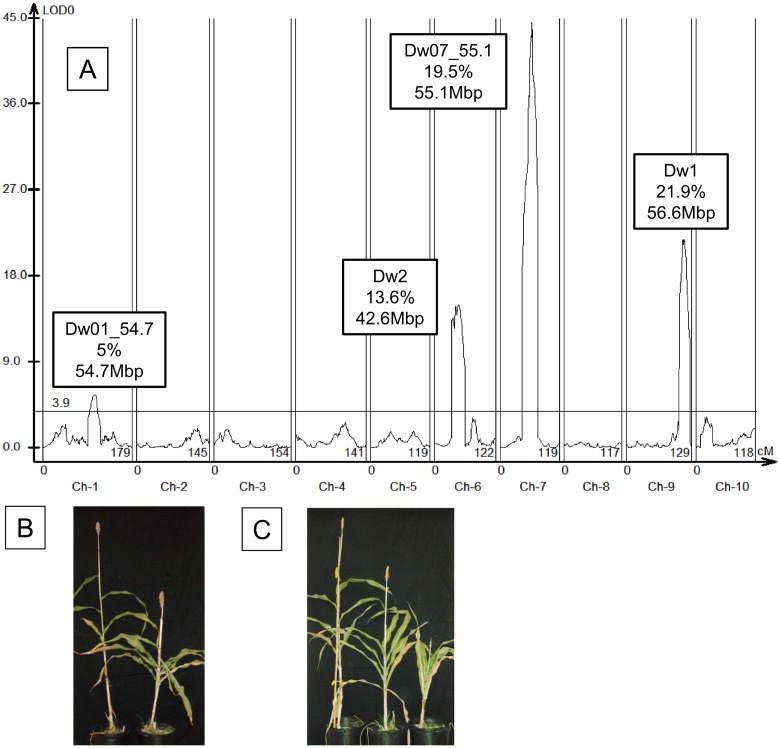
Stem internode length QTL identified in a population from Hegari x 80M. F_2_ plants from a cross of Hegari and 80M (n = 218) were grown in the greenhouse and the length of each internode was measured. The average internode length was used to map QTL. (A) The resulting graph shows four QTL, including *Dw1* and *Dw2*. The x-axis is the genetic map and the y-axis is the LOD score. The boxes above each trait identify the *Dw* loci, if any, the percentage of the variation explained by the QTL, and the location of the peak LOD value. (B) Photograph of Hegari (left) and 80M. (C) Photograph of F_5_ plants that are *Dw1Dw1* (left), *Dw1dw1* (center), and *dw1dw1* (right) in otherwise uniform genetic backgrounds at the other loci that affect internode length.

An F_2_ population for mapping *Dw1* was constructed by crossing Hegari (*Dw1*) and 80M (*dw1*). The F_2_ population segregated for flowering time and height. The length of expanded internodes was measured for all plants in the population with the first expanded internode being labeled as number 5. Four QTL were identified that modulate the average length of internodes 5–10 (Fig 1, Table 1). A QTL corresponding to *Dw1* was identified on SBI-09 with a peak at ~56.6 Mbp on *Sorghum bicolor* genome v2 (Phytozome v10). This QTL explained ~22% of the trait variance observed. The *Dw1* allele in Hegari increased the lengths of all expanded internodes compared to plants containing the *dw1* allele present in 80M (S1 Fig). A second QTL for internode length was located on SBI-06 at ~42.6 Mbp that aligned with *Dw2* [15]. A previously reported QTL for internode length was identified on SBI-01 at ~54.7 Mbp (*Dw01_54*.*7*) that explained ~5% of the variance [41,42]. A QTL on SBI-07 at ~55.1 Mbp (*Dw07_55*.*1*) that was recently described by Li et al [16] explained 19% of the variance. The QTL on SBI-07 (*Dw07_55*.*1*) was 3 Mbp from the *ABCB1* gene corresponding to *Dw3* (58.6 Mbp). No QTL aligned with *ABCB1* as expected because both parental genotypes are *Dw3*.

**Table 1:** QTL for Average Internode Length Identified in the Entire Population of Hegari x 80M F_2_.

QTL	Chr	Peak (cM)	Peak LOD	Peak (Mbp)	Additive*	Dominance*	R^2^	Dw locus
1	1	104.2	5.53	54.7	12.5848	-5.5165	0.0503	*Dw01_54*.*7*
2	6	46.5	15	42.6	-22.8162	4.1926	0.1358	*Dw2*
3	7	62.4	44.37	55.1	39.2763	22.2605	0.1945	*Dw07_55*.*1*
4	9	112.2	21.8	56.6	-27.3763	6.4375	0.2186	*Dw1*

* Positive means the allele from 80M increases length; negative is Hegari.

QTL mapping was also performed using data on fresh and dry weight per internode, fresh or dry weight per unit stem length, and diameter of internode 7 (S4 Table). Alleles of *Dw1* contributed to variation for internode fresh weight and dry weight.

### Analysis of Epistasis

Potential interactions among the four QTL modulating internode length were investigated using multiple-QTL mapping in R/qtl [27]. The best model (y ~ *Dw01_54*.*7* + *Dw2* + *Dw07_55*.*1*+ *Dw1* + *Dw10_3*.*2*+ *Dw07_55*.*1*:*Dw1*) had a pLOD of 50.1 and included five QTL and an interaction between two of the QTL (*Dw1* and *Dw07_55*.*1*, S5 Table). The analysis showed an interaction between *Dw1* and *Dw07_55*.*1* such that allelic variation in *Dw1* has minimal impact on internode length in the presence of the 80M allele at *Dw07_55*.*1* which increased internode length (S2 Fig). In addition, the 80M allele of *Dw07_55*.*1* increased internode length in *Dw1Dw1*, *Dw1dw1*, and *dw1dw1* backgrounds, although to a greater extent in genotypes that were *dw1dw1*. These results indicate that *Dw1* and *Dw07_55*.*1* independently activate the same downstream regulator of internode elongation, or act through different pathways to stimulate internode growth.

### Fine Mapping *Dw1*

**Fig 2:**
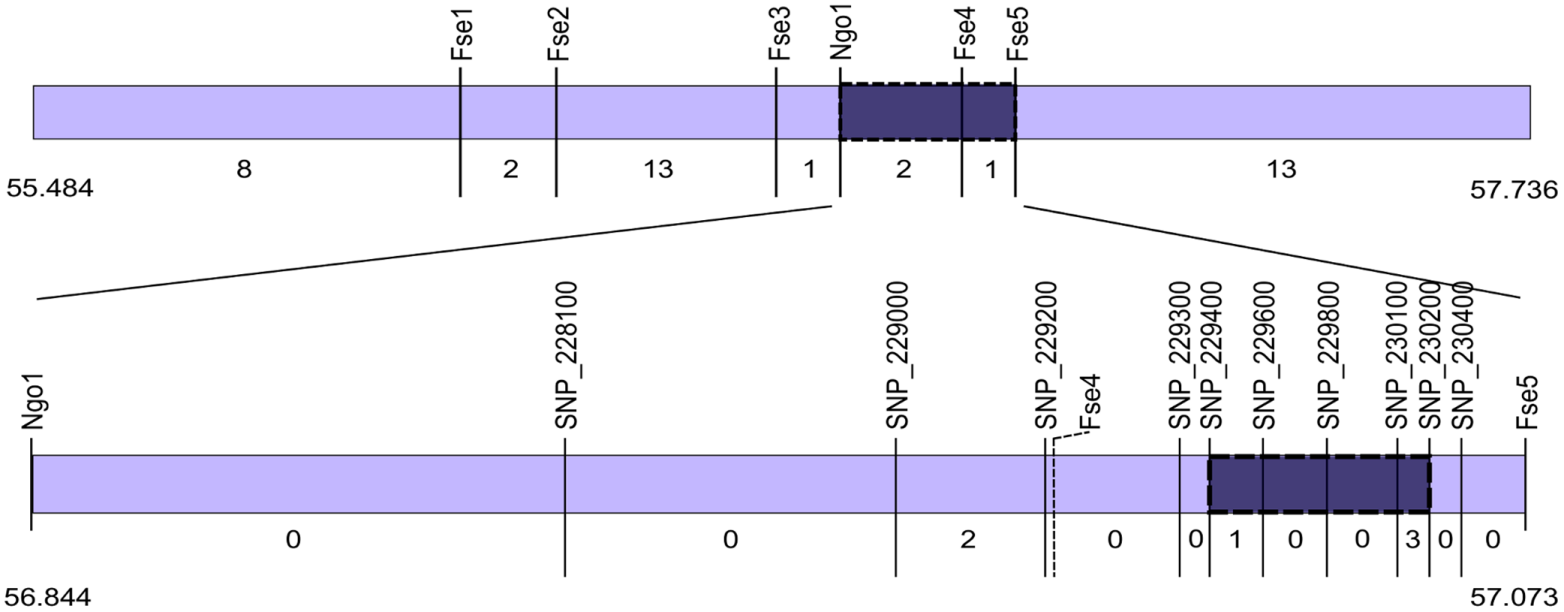
A schematic of the region of SBI-09 encoding *Dw1*. The top bar shows the *Dw1* locus delimited by QTL mapping in the F_2_. The region was refined in the F_3_ population (n = 75 for each of six families) using the DG markers labeled in the diagram. The numbers below the bar are the number of recombinants (both bars). Note that all members of one of the families (237) had a breakpoint in between Fse5 and the end of the region shown. The lower bar represents the delimited *Dw1* locus defined by mapping in the F_3_ generation with SNP markers labeled. Dark purple shows the location of *Dw1* based on fine mapping. SNP markers are named with the last six digits of the gene name of the gene the SNP is in or near. Fse4 is included for perspective though it was not scored in the F_4_.

*Dw1* was fine mapped by constructing HIFs from seed of F_2_ plants of the QTL mapping population that were heterozygous for *Dw1* and homozygous at the other QTL that affect internode length. HIFs derived from F_2_ plants homozygous for the Hegari allele at *Dw07_55*.*1* were most useful for fine mapping *Dw1*. F_3_ individuals from each HIF family were grown in the greenhouse until grain maturity, and then stems were phenotyped for internode length. Histograms of the average internode length for each HIF are shown in S3 Fig. Breakpoint analysis of the first set of HIFs narrowed the region encoding *Dw1* to 313kb. The location of breakpoints in a few key lines was further refined using Digital Genotyping based on the restriction enzyme NgoMIV [24]. This information delimited the *Dw1* locus to 230kb, a region encoding 35 genes as annotated in the v1.4 gene set (Phytozome v.9). A further round of fine mapping was carried out using five HIFs derived from F_3_ plants heterozygous for *Dw1dw1*. These plants were screened for recombinants with CAPS markers and six plants were identified with recombination breakpoints in the delimited *Dw1* region. Phenotyping and identification of breakpoints by sequencing SNPs delimited *Dw1* to a region that spanned 33kb and encoded seven genes as annotated in v2.1 (Phytozome v.10) (Table 2). Markers used for fine mapping and the location of the delimited *Dw1* locus are shown in Fig 2. Information about the seven putative genes in the delimited *Dw1* locus is provided in Table 2. Four of the genes were annotated with a function: an E3-ubiquitin ligase involved in syntaxin degradation, Photosystem I reaction center subunit VI, PRONE-Rop nucleoide exchanger, and a serine/threonine kinase. There were also three genes annotated as having unknown functions.

**Table 2:** Genes in the Delimited *Dw1* Locus.

Gene Name	Probable Function	Location
Sobic.009G229500	Unknown	57,026,900–57,027,289
Sobic.009G229600	E3 ubiquitin ligase/syntaxin degradation	57,027,335–57,036,566
Sobic.009G229700	Photosystem I reaction center, subunit VI	57,036,793–57,037,995
Sobic.009G229800	Unknown	57,042,620–57,045,133
Sobic.009G229900	PRONE-Rop nucleotide (guanine) exchanger	57,046,394–57,049,526
Sobic.009G230000	Unknown	57,050,065–57,051,463
Sobic.009G230100	Serine/threonine kinase	57,051,814–57,055,008

### Identification of Polymorphisms in the Delimited *Dw1* locus

**Fig 3:**
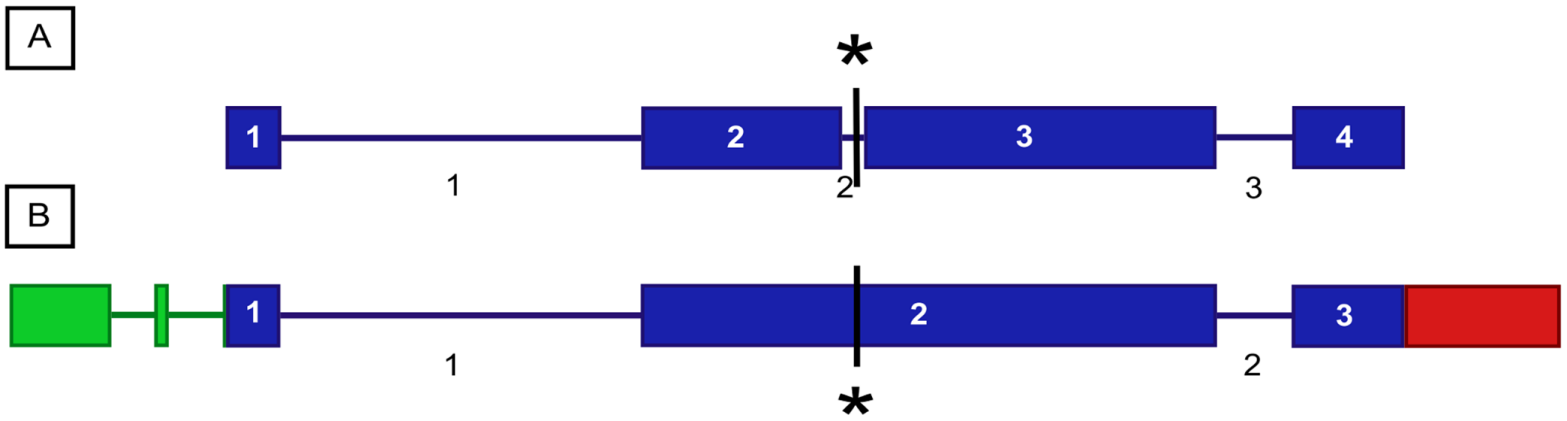
Gene annotation models of *Dw1* (Sobic.009G229800). (A) Gene model from *Sorghum bicolor* Genome v2.1 (Phytozome). (B) Gene model based on cDNA sequence analysis. Boxes (blue) represent exons and lines are introns. Regions colored green represent the 5’ UTR and those colored red the 3’ UTR. Exons are numbered within boxes and introns are numbered in black. The asterisk/vertical line marks the location of the Lys199 > stop codon mutation that distinguishes *Dw1* from *dw1*.

All seven genes located in the fine mapped *Dw1* locus were sequenced in Hegari and 80M (Table 3). No sequence variants were found in Sobic.009G229700 or Sobic.009G229900. The only sequence variants in Sobic.009G229600 and Sobic.009G230100 were located in introns and/or the 5’UTR. Of the genes annotated with an unknown function, Sobic.009G229500 had no sequence variants while Sobic.009G230000 had two INDELs in the 5’UTR and a SNP in the first exon that resulted in a synonymous mutation. Sobic.009G229800 was the only gene in the delimited *Dw1* locus that had a polymorphism distinguishing the parental genotypes that resulted in a change in amino acid sequence (Table 3). Hegari (*Dw1*) encoded a full-length protein, whereas the sequence in 80M (*dw1*) (and BTx623 (*dw1*)) contained an A > T mutation that caused a Lys199 > stop codon change in the second exon of Sobic.009G229800 (Fig 3B).

**Table 3:** Polymorphisms Distinguishing 80M and Hegari in Genes in the Delimited *Dw1* Locus.

Gene	Number	Type	Polymorphism	Location	Region
**Sobic.009G229500**	None				
**Sobic.009G229600**	1	SNP	C > T	2660	Intron
	2	INDEL	- > A	6597	Intron
**Sobic.009G229700**	None				
**Sobic.009G229800**	1	INDEL	A > -	-707	5' UTR
	2	SNP	A > T; K > Stop	1350	Exon
**Sobic.009G229900**	None				
**Sobic.009G230000**	1	INDEL	- > CAGGCAGG	-64	5'UTR
	2	INDEL	- > ACGACG	-25	5'UTR
	3	SNP	G > T; L > L	126	Exon
**Sobic.009G230100**	1	INDEL	T > -	-397	5' UTR
	2	SNP	A > T	537	Intron
	3	INDEL	A > -	1841	Intron

All seven of the genes in the delimited region were also sequenced in Standard Yellow Milo (*Dw1*) and Dwarf Yellow Milo (*dw1*). Quinby [23] noted that *dw1* was originally identified in the Standard Yellow Milo (*Dw1*, *Dw2*, *Dw3*) background [43]. The shorter version of Yellow Milo containing *dw1* was named Dwarf Yellow Milo. Therefore, the sequences of Standard Yellow Milo and Dwarf Yellow Milo are expected to vary only at *Dw1*. Sequence analysis revealed only one polymorphism in the delimited *Dw1* region that distinguished the two milo lines: the A > T SNP in Sobic.009G229800 that caused a premature stop codon. For all the other polymorphisms found between Hegari and 80M in the region, Standard Yellow Milo and Dwarf Yellow Milo had the same allele as 80M.

The gene-model for Sobic.009G229800 in v2.1 (Phytozome v10) included a very short intron (intron 2) (Fig 3A). However, cDNA sequence analysis of Sobic.009G229800, and RNA-seq analysis (see below), failed to provide evidence for intron 2. Instead, cDNA sequences from Hegari (*Dw1*) contain a continuous coding region that spanned intron 2 of the v2.1 gene-model. Gene-models of homologs of Sobic.009G229800 in other plant species (e.g. maize, rice, and Arabidopsis) also lack intron 2 and show continuous reading frames across this region. The cDNA sequence also clarified splicing in the 5’UTR (Fig 3, regions in green). Based on this analysis, we propose the revised annotation of Sobic.009G229800 shown in Fig 3B that contains three exons and conclude that the polymorphism that distinguishes Hegari and 80M generates a truncated protein lacking most of exon 2 and all of exon 3 (mutation marked by an asterisk in Fig 3) presumably resulting in a loss of function.

The intron/exon structures of the other genes in the delimited *Dw1* locus were identical to homologs in maize and/or rice (S6 Table). Furthermore, the RNA-seq data for v3.1 (Phytozome v11) is consistent with the annotations of the other genes in the delimited *Dw1* locus and the updated annotation of Sobic.009G229800 that lacks intron 2 (Fig 3B).

Sobic.009G229800 was sequenced in other genotypes of sorghum previously identified as *Dw1* or *dw1* (Tables 4 and 5; S7 Table). Genotypes previously designated as *Dw1* encoded full-length proteins similar to Hegari. Numerous grain sorghum-breeding lines with shorter internodes were generated from the Dwarf Yellow Milo source of *dw1*. Therefore, it is not surprising that all of the lines designated *dw1* have the same recessive allele as Dwarf Yellow Milo. Sobic.009G229800 sequences from Rio and Early White Milo (both *Dw1*) contain several additional polymorphisms (Table 5). SIFT [44] analysis of a non-synonymous coding mutation found in Rio and Early White Milo (A425S) predicted that this change in *Dw1* would not affect function.

**Table 4:** Sequence Variants in Exons of Sobic.009G229800 in Diverse Sorghum Genotypes.

Number	2	13	14	15	16	17	18	19	22
**Polymorphism**	A > T	C > T	G > A	G > A	C > A	T > C	T > A	T > G	T > C
Location (bp)*	1350	1127	1259	1583	1586	1667	1733	2028	2316
**Region**	Exon 2	Exon 2	Exon 2	Exon 2	Exon 2	Exon 2	Exon 2	Exon 2	Exon 3
**Type**		Syn	Syn	Syn	Syn	Syn	Syn	Nonsyn	Syn
**Change in Protein**	K > Stop	F > F	P > P	S > S	P > P	T > T	P > P	S > A	N > N
**SIFT**	N/A	N/A	N/A	N/A	N/A	N/A	N/A	0.36 = tolerated	N/A

*From the start codon

**Table 5:** Distribution of *Dw1* Coding Sequence Variants in Sorghum Genotypes. The polymorphism number corresponds to the number in Table 4.

Line	*Dw1* Genotype	Polymorphism Number								
		2	13	14	15	16	17	18	19	22
Hegari	*Dw1*	A	T	A	A	A	C	A	G	C
80M	*dw1*	T	T	A	A	A	C	A	G	C
Standard Yellow Milo	*Dw1*	A	T	A	A	A	C	A	G	C
Dwarf Yellow Milo	*dw1*	T	T	A	A	A	C	A	G	C
Double Dwarf Yellow Milo	*dw1*	T	T	A	A	A	C	A	G	C
BTx623	*dw1*	T	T	A	A	A	C	A	G	C
BTx406	*dw1*	T	T	A	A	A	C	A	G	C
SC170	*dw1*	T	T	A	A	A	C	A	G	C
R.07007	*dw1*	T	T	A	A	A	C	A	G	C
IS3620c	*dw1*	T	T	A	A	A	C	A	G	C
Rio	*Dw1*	A	C	G	G	C	T	T	T	T
M35-1	*Dw1*	A	T	A	A	A	C	A	G	C
Texas Blackhull Kafir	*Dw1*	A	T	A	A	A	C	A	G	C
Spur Feterita	*Dw1*	A	T	A	A	A	C	A	G	C
Early White Milo	*Dw1*	A	C	G	G	C	T	A	T	T

### Expression of *Dw1* in Stem Tissue

**Fig 4:**
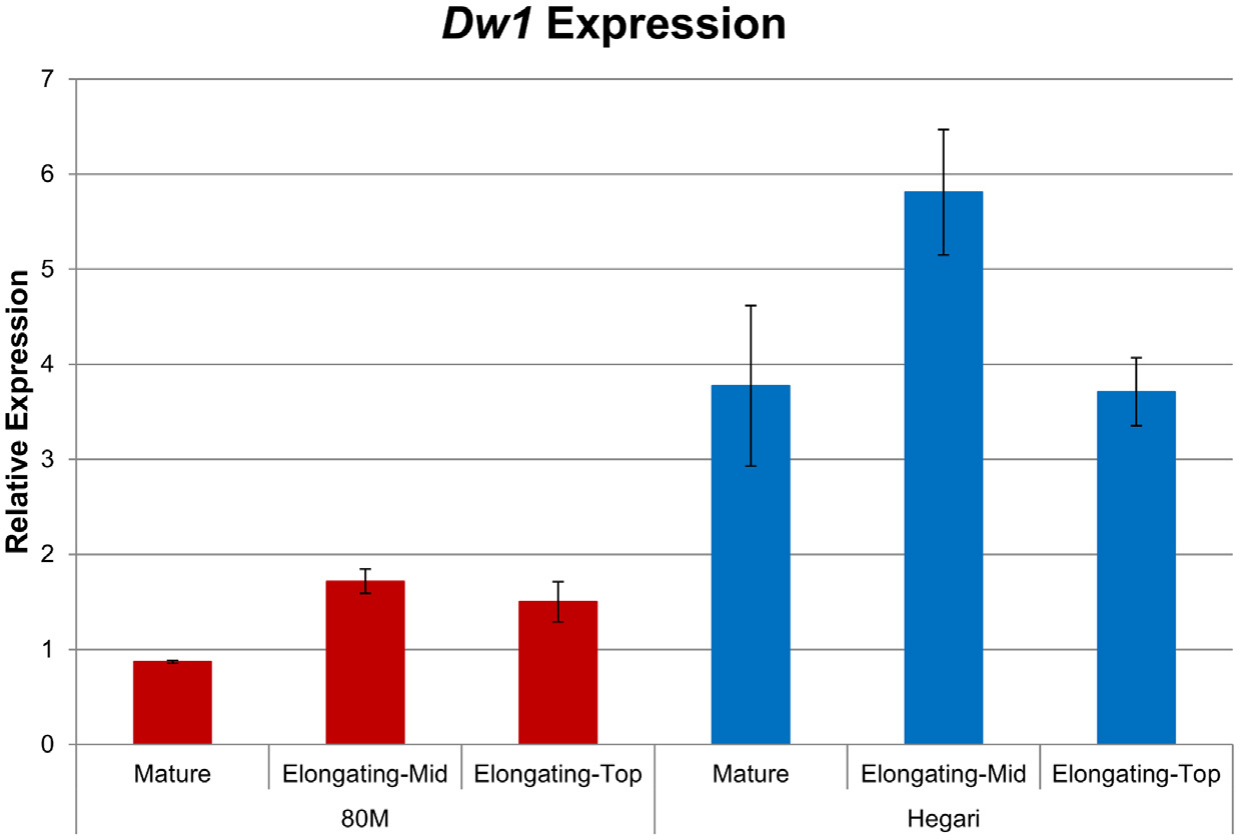
Relative expression of *Dw1* in stem internodes. RNA was extracted from a full length internode (Mature), the lower half of an elongating internode, and the upper half of an elongating internode for each parental genotype (n = 3 each). Relative expression was determined by qRT-PCR using the ΔΔCt method with 18S rRNA as the normalizer and the sample from 80M mature tissue as the calibrator.

Sobic.009G229800 was expressed in fully elongated internodes and elongating internodes (Fig 4). The highest levels of expression were observed in the lower portion of the elongating internode. *Dw1* mRNA levels were ~3-fold higher in stems of Hegari compared to 80M.

### Protein Sequence Analysis

**Fig 5:**
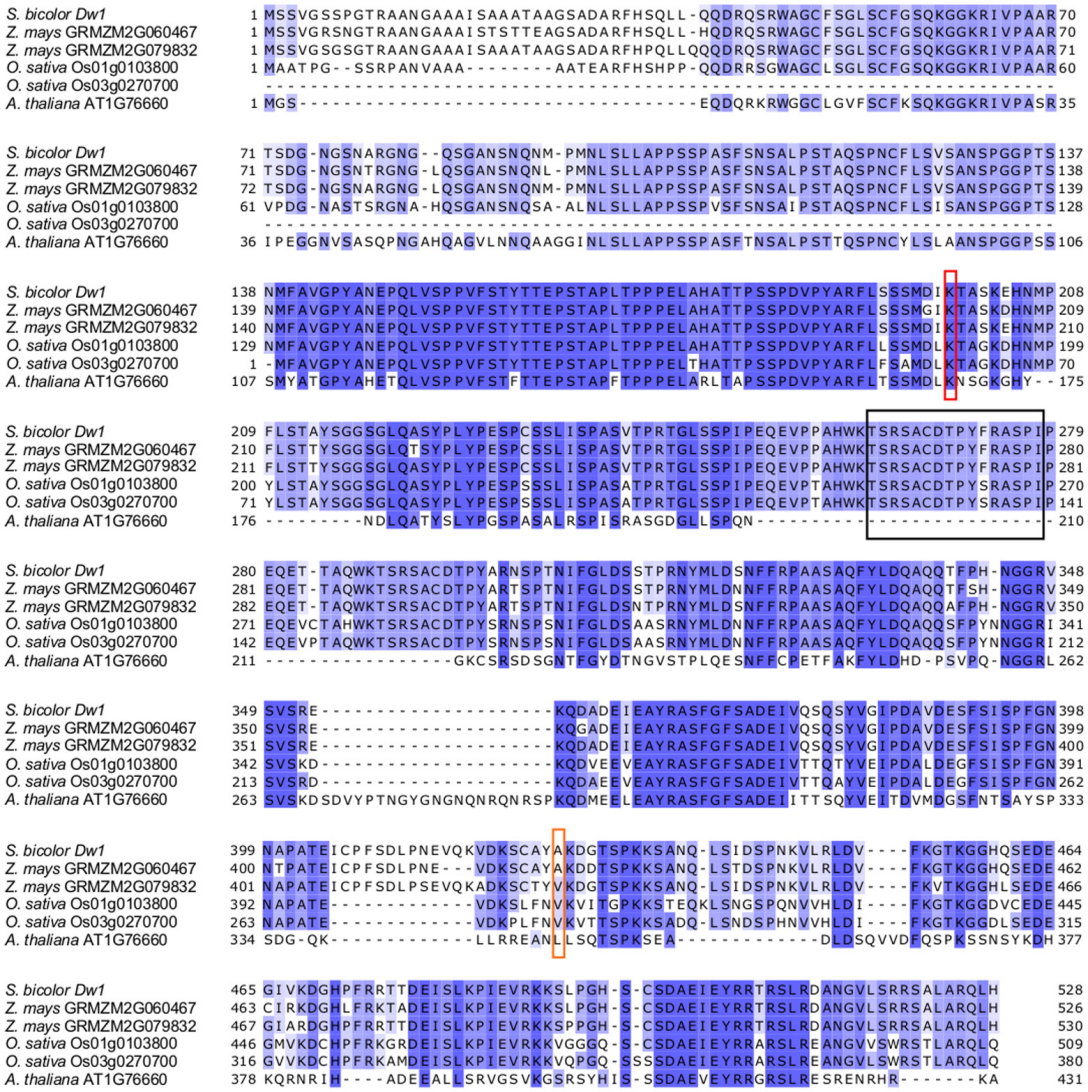
Protein alignment of *Dw1* and select homologs. Alignment of *Dw1* with the two maize homologs, the two rice homologs, and the Arabidopsis homolog compiled in Jalview using the T-Coffee function (dark blue color indicates higher percent identity). The red rectangle marks the functional polymorphism that distinguishes Hegari (*Dw1*) and 80M (*dw1*). The orange rectangle marks a polymorphism present in Rio and Early White Milo not found in the other sequenced lines. The black box is the possible transmembrane domain predicted by PSIPRED-MEMSAT-SVM.

Sobic.009G229800 is currently annotated as having an unknown function. BLAST analysis showed that homologous genes/proteins are present in maize, rice, and Arabidopsis among other plants (S8 and S9 Tables). Fig 5 shows the sequence alignment of Sobic.009G229800 and maize, rice, and Arabidopsis homologs. A phylogenetic tree of select homologs has two distinct groups corresponding to the monocots and dicots (S4 Fig). The Arabidopsis homolog of *Dw1* is annotated as associated with the plasma membrane based on experimental evidence [45] and located in the nucleus based on prediction (TAIR). PSIPRED-MEMSAT-SVM predicts that the sorghum Dw1 protein contains a single transmembrane/pore-lining domain from residues 263–278. Interestingly, these residues are missing in the Arabidopsis homolog (Fig 5). PSIPRED-DISOPRED, PONDR, and FoldIndex all predicted a high degree of disorder in the protein (S10 Table).

Maize homologs of Sobic.009G229800 located on chromosomes 6 and 8 are syntenic to sorghum chromosome 9. Genes flanking *ZmDw1* on maize chromosome 8 show collinearity with the region on SBI-09 encoding *Dw1*. On the other hand, the *OsDw1* homologs are located on rice chromosomes 1 and 3 while sorghum chromosome 9 is syntenic to rice chromosome 5. This suggests that *Dw1* moved to its position on SBI-09 after separation from rice and before separation from maize.

## Discussion

In this study, *Dw1* was identified using a F_2_ population and HIFs derived from Hegari (*Dw1*) and 80M (*dw1*). *Dw1* was identified as Sobic.009G229800 a gene of unknown function that is highly conserved in plants. The recessive *dw1* allele corresponds to a loss of function mutation that creates a stop codon in the middle of the protein encoded by Sobic.009G229800. The recessive *dw1* allele identified in 80M was present in Dwarf Yellow Milo (*dw1*) and Double Dwarf Yellow Milo (*dw1*,*dw2*) but not in Standard Yellow Milo (*Dw1*) consistent with reports that short plants containing *dw1* originated as a spontaneous mutation in Standard Yellow Milo [6,23]. 80M and the other maturity standards (i.e., 100M, 90M, 80M, 60M) were derived from a cross of Early White Milo (*Dw1*) and Double Dwarf Yellow Milo (*dw1*, *dw2*) and progeny recessive for *dw1* and *dw2* were selected so that the maturity standards have similar internode lengths (*dw1dw2Dw3dw4*) [23].

The Dwarf Yellow Milo *dw1* allele is present in BTx623, an elite seed parent, and in other genotypes used for grain sorghum breeding in the U.S. (i.e., BTx406, SC170, R07007). The *dw1* allele described in this study is present in many grain sorghum lines because BTx406 (*dw1*) was used to convert tall late flowering sorghum accessions to short early flowering genotypes useful for grain sorghum breeding in the U.S. [15]. This also explains why Brown et al. [14] mapped a QTL for height (*Sb_HT9*.*1*) corresponding to allelic variation at the *Dw1* locus in a panel of grain genotypes many of which included BTx406 in their pedigrees. Markers most tightly linked to *Sb_HT9*.*1* identified a region of SBI-09 from 57.14–57.21, the same region we found that encodes *Dw1*. This region includes Sobic.009G229800; however, this gene was initially annotated in Phytozome as two genes (v1.4 gene set). Subsequently, Sobic.009G229800 was annotated with an intron spanning the portion of the coding region that contains the causative mutation (v2.1). Two additional mapping studies identified the same region of SBI-09 as encoding *Dw1* [20,21]. Both studies suggested that mutations in a GA2 oxidase (GA2ox5) could be responsible for variation in height caused by *Dw1*. However, subsequent sequence analysis of *GA2ox5* from genotypes that were *Dw1Dw1* and *dw1dw1* did not show sequence variants consistent with the identification of this gene as *Dw1* [22]. Moreover, mutations causing reduced GA levels in sorghum result in short internodes but also abnormal culm bending, a phenotype not observed in *dw1dw1* sorghum genotypes [22].

*Dw1* (Sobic.009G229800) is present in maize, rice, other grasses, and dicots such as Arabidopsis. Several large INDELS distinguish the proteins in grasses and Arabidopsis. Homologs of Sobic.009G229800 in maize are collinear with *Dw1* in sorghum; however, homologs in rice are not located on the homeologous chromosome suggesting that this gene moved to its current location in sorghum after separation of these grasses. The closest homolog in Arabidopsis is annotated as a plasma membrane protein, a localization that was verified experimentally [45]. The Arabidopsis protein was also annotated with a nuclear location. Analysis of the sorghum protein identified a stretch of amino acids (263–278) that could be associated with the lining of a transmembrane pore. The protein was also predicted to have highly disordered protein domains. Research clarifying the localization and biochemical function of the protein encoded by Sobic.009G229800 will be needed to understand how *Dw1* regulates the length of stem internodes.

Quinby and Karper [46] showed that alleles of *Dw1* do not affect leaf size, only internode lengths. The restriction of *Dw1* action to stems is useful because *dw1dw1* can be used to reduce internode length without affecting leaf morphology or canopy development. Furthermore, a QTL corresponding to *Dw1* was also found to modulate the weight of the stem but not weight per unit length of stem. Thus, *Dw1* increases length and weight of internodes. Heterozygous *Dw1dw1* progeny derived from Hegari x 80M had internode lengths that were intermediate compared to plants that were *dw1dw1* and *Dw1Dw1* (S2 Fig), indicating gene dosage alters the gene’s action on internode growth. *Dw1* was expressed in stem internodes, with ~3-fold higher expression in Hegari (*Dw1*) compared to 80M (*dw1*). Higher expression in Hegari could be due to feedback from *Dw1* resulting from greater growth of the internode, or due to differences in Hegari/80M genetic background.

This research was undertaken to further our understanding of genetic factors influencing internode elongation and stem length in sorghum with a focus on *Dw1*. QTL analysis of an F_2_ population derived from Hegari and 80M used for fine mapping *Dw1* identified QTL that modulate stem internode length aligned with *Dw1*, *Dw2*, a minor QTL on SBI-01 (*Dw01_54*.*7*) and a QTL on SBI-07 approximately 3Mbp from *Dw3* (*Dw07_55*.*1*) [16]. Interactions between *Dw07_55*.*1* and *Dw1* were detected and plants homozygous for the *Dw07_55*.*1* allele from 80M had long internodes and showed attenuated influence of *Dw1* alleles in this background. *Dw3* is an ABCB1 efflux auxin transporter that has homologs in many other plants. However, the phenotypic effect of mutation of ABCB1 is attenuated in dicots like Arabidopsis where auxin is exported from apical meristems via two different ABCB transporters: ABCB1 and ABCB19 [47]. In grasses, auxin is exported from the apical meristem and intercalary meristems of the stem. ABCB1 in maize is the only ABCB transporter in the intercalary meristem leading to more severe stem internode length phenotypes when this gene is mutated. Interestingly, in maize the ABCB1 mutant causes severe shortening of the lower internodes while the upper internodes are essentially normal in length [13]. In contrast, *dw1dw1* caused a reduction in the length of all internodes (S1 Fig). The current study and prior studies showed that recessive *dw1* alleles decrease internode length/plant height in *Dw3* backgrounds (Standard Yellow Milo, Dwarf Yellow Milo) as well as in plants that are homozygous for *dw3* (Texas Blackhull Kafir (*Dw1Dw2dw3*) vs Martin (*dw1Dw2dw3*) [6]. This result suggests that *Dw1* action is not dependent on *Dw3*, although *Dw3* alleles may modulate the extent of *Dw1* action on internode elongation. As noted above, *Dw1* is not a GA2 oxidase as previously suggested and recessive alleles do not result in stem bending. However, it is possible that *Dw1* mediates signaling by hormones (GA, auxin, brassinosteroids, strigolactone, ethylene), photoreceptors (phytochromes, PIFs), or other factors that modulate internode growth. Ongoing research is focused on characterizing the molecular basis of *Dw1* action.

## Supporting Information

S1 Fig Internode length versus internode number for a HIF. The average internode length for each internode was calculated for each genotype at *Dw1* for one of the F_3_ HIFs (n = 75). In (A) the internodes are numbered from the bottom of the stem, whereas in (B) they are numbered from the peduncle.
(TIFF)

S2 Fig Interaction plots from MQM mapping in R/qtl. The interaction plots show the interaction between *Dw1* and the locus on chromosome 7 (*Dw07_55*.*1*) in the Hegari x 80M F_2_. The A allele is 80M and the B allele is Hegari. Phenotypes distinguishing *Dw1* from *dw1* are greater when the *Dw07_55*.*1* locus on LG-07 is BB (fixed Hegari).
(TIFF)

S3 Fig Histograms of the average internode length for each Hegari x 80M F_3_ HIF. For each HIF, the lines that had recombination break points in the region of *Dw1* were removed and the remainder of the plants grouped into *Dw1Dw1* (blue), *Dw1dw1* (red), and *dw1dw1* (green) and plotted in a histogram. Note that HIFs 74 and 237 have the 80M allele at *Dw7_55*.*1* while the others have the Hegari allele.
(TIFF)

S4 Fig A phylogenetic tree of a diverse selection of *Dw1* homologs. Tree was constructed in MEGA6 using Maximum Likelihood. Sorghum *Dw1* is in bold letters.
(TIFF)

S1 Table Primers for Fine Mapping. (DOCX)

S2 Table Primers for Sequencing of Candidate Genes. (DOCX)

S3 Table Primers for Amplifying cDNA of Sobic.009G229800. (DOCX)

S4 Table *Dw1* QTL for Each Trait for Hegari x 80M F_2_. (DOCX)

S5 Table QTL for Average Internode Length Identified using MQM in R/qtl. (DOCX)

S6 Table Maize and Rice Homologs of the Seven Genes in the Delimited *Dw1* Region. (DOCX)

S7 Table All Polymorphsims of Sobic.009G229800 in Selected Genotypes. The number in the polymorphism table is the same as in the genotypes scored table. The numbers also correspond to the numbers in Tables 4 & 5.
(XLSX)

S8 Table Homologs of *Dw1* (NCBI-top 100 hits). Accessions included in the phylogenetic tree are bolded.
(XLSX)

S9 Table Homologs of Sobic.009G229800 (Phytozome). (XLSX)

S10 Table Summary of Protein Function Searches. (DOCX)
