# Supplementary figures and images for: Identification of Dw1, a Regulator of Sorghum Stem Internode Length
Source: PLoS One. 2016 Mar 10;11(3):e0151271. doi: 10.1371/journal.pone.0151271 (PMC4786228; doi:10.1371/journal.pone.0151271)

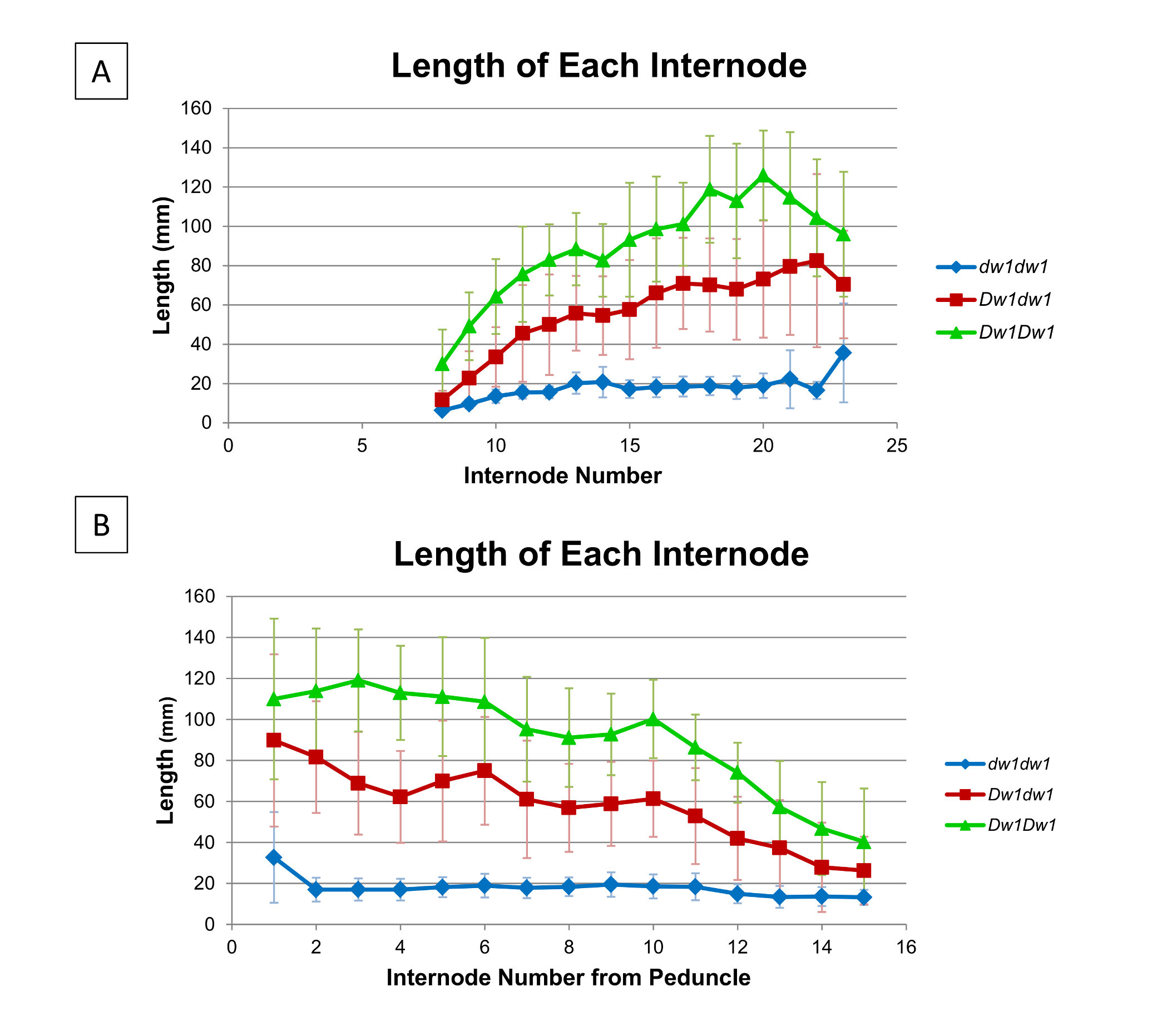

Supplement: S1 Fig — The average internode length for each internode was calculated for each genotype at Dw1 for one of the F3 HIFs (n = 75). In (A) the internodes are numbered from the bottom of the stem, whereas in (B) they are numbered from the peduncle. (TIFF) [file pone.0151271.s001.tiff]

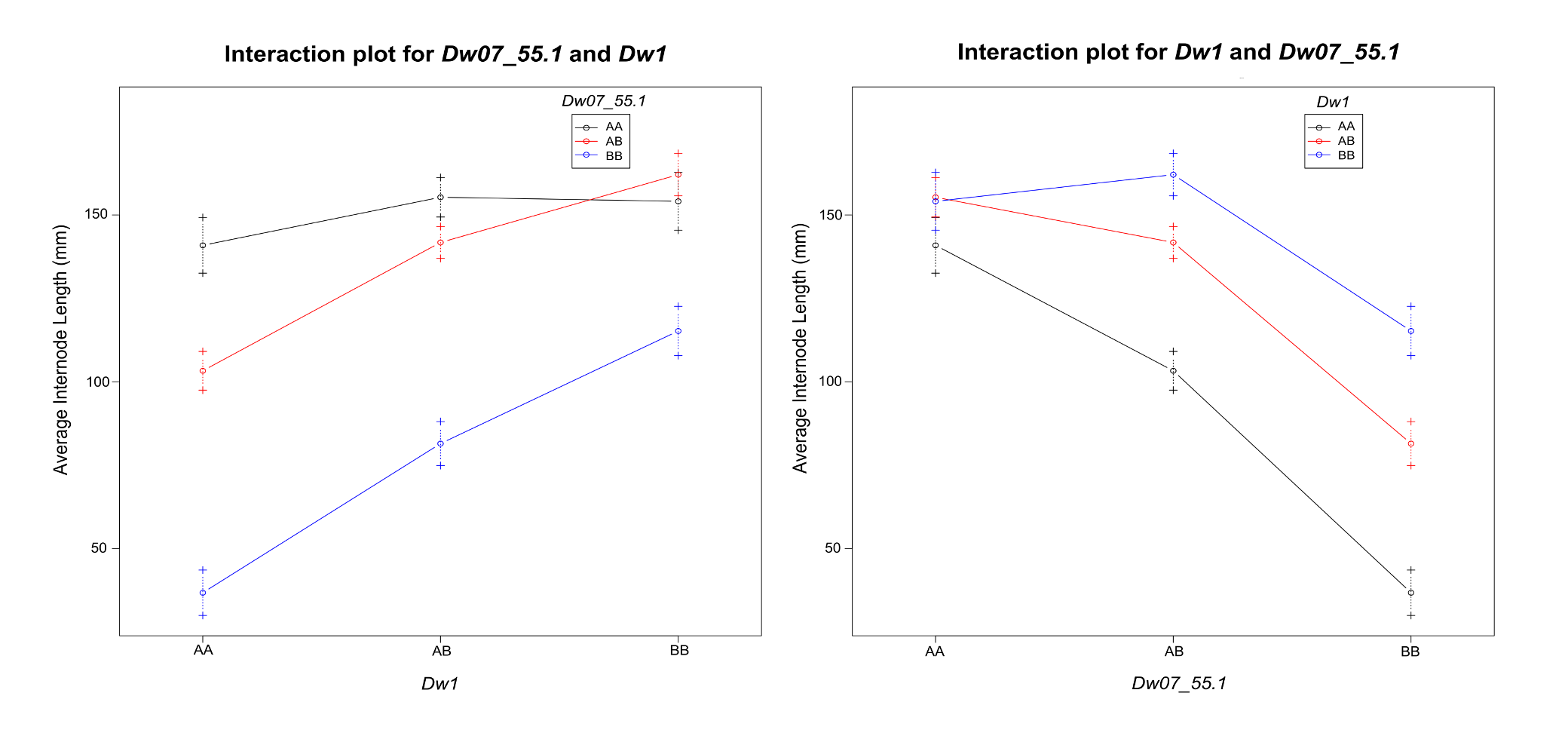

Supplement: S2 Fig — The interaction plots show the interaction between Dw1 and the locus on chromosome 7 (Dw07_55.1) in the Hegari x 80M F2. The A allele is 80M and the B allele is Hegari. Phenotypes distinguishing Dw1 from dw1 are greater when the Dw07_55.1 locus on LG-07 is BB (fixed Hegari). (TIFF) [file pone.0151271.s002.tiff]

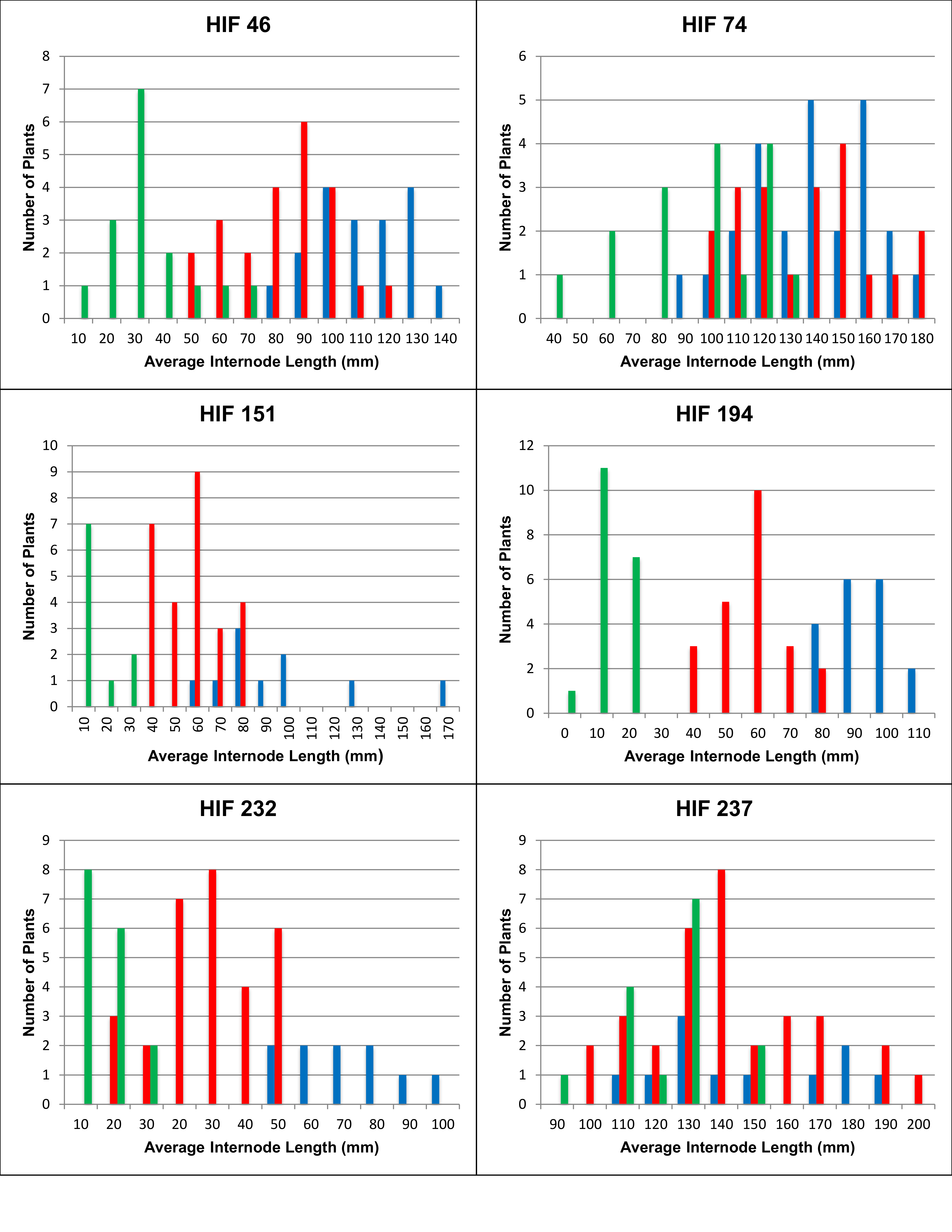

Supplement: S3 Fig — For each HIF, the lines that had recombination break points in the region of Dw1 were removed and the remainder of the plants grouped into Dw1Dw1 (blue), Dw1dw1 (red), and dw1dw1 (green) and plotted in a histogram. Note that HIFs 74 and 237 have the 80M allele at Dw7_55.1 while the others have the Hegari allele. (TIFF) [file pone.0151271.s003.tiff]

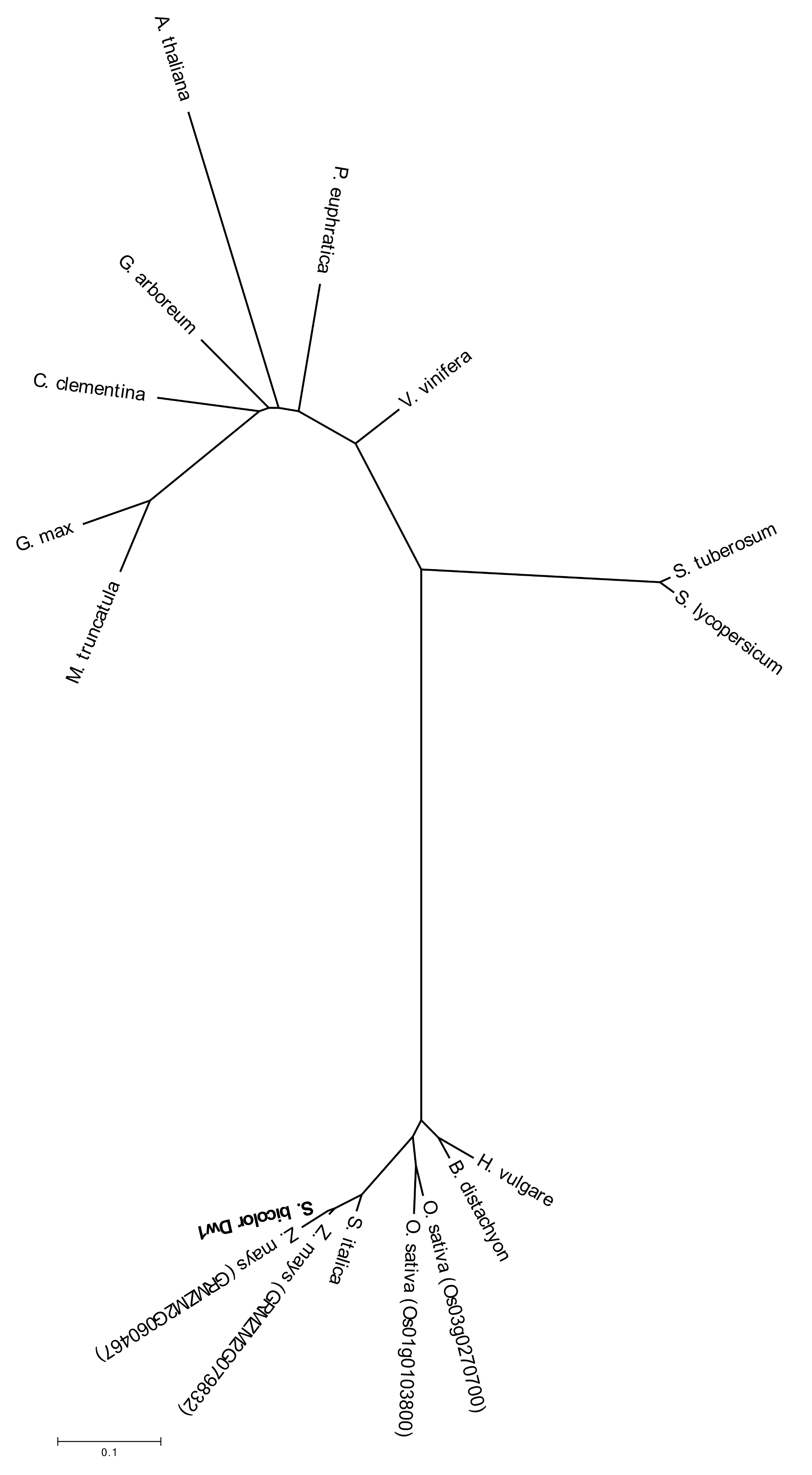

Supplement: S4 Fig — Tree was constructed in MEGA6 using Maximum Likelihood. Sorghum Dw1 is in bold letters. (TIFF) [file pone.0151271.s004.tiff]
