# Supplementary material for: Identification of Dw1, a Regulator of Sorghum Stem Internode Length
Source: PLoS One. 2016 Mar 10;11(3):e0151271. doi: 10.1371/journal.pone.0151271 (PMC4786228; doi:10.1371/journal.pone.0151271)
Supplement: S1 Table — (DOCX) [file pone.0151271.s005.docx]

| **Marker Name** | **Forward Primer Sequence** | **Reverse Primer Sequence** | **Marker type** | **RE (CAPS only)** | **Location (bp)** |
| --- | --- | --- | --- | --- | --- |
| CAPS_2 | GGCAAGCTTAGTTGAAGTTGTT | GTCCCAATGACTTGGCTATCT | CAPS | CviQI | 56,763,365 |
| SNP_110 | CAAGGTTTCTCTGCCACTAGAC | TGCTTGGGTAACGTGGTAATC | SNP |  | 56,925,217 |
| SNP_180 | GGTGTTTCATCGTCCTCCTATC | CCGTACCTGATGATGGGATTAG | SNP |  | 56,975,859 |
| SNP_210 | CGGGTGGCAATTAGAAGTAAGG | TCCATCCATGCTGACCTTAAC | SNP |  | 56,998,902 |
| SNP_220 | TGCTCCTGAAACTGCTAACC | GAGGAGGTTCCAGGTTGAAAT | SNP |  | 57,020,309 |
| SNP_230 | TGTTTAGGAAGGCTCCATGTC | TCCAACGCCACAACTGTAA | SNP |  | 57,025,222 |
| SNP_250 | GATCGCCTAACAGCATGTAATTC | TACCCTACGCATGAGGATAAGA | SNP |  | 57,033,287 |
| SNP_270 | GGACTAACACACGCTTCTCTAC | CATCTTGCTTCTCCCTGGATAC | SNP |  | 57,043,969 |
| SNP_300 | TCTGATGCGACCGATCTTTG | CCTGAAGCAGGTCTCTGAATG | SNP |  | 57,053,808 |
| SNP_310 | CCATGCACATGGTCGTTATG | GGACGTACTCGTAGCTGAAG | SNP |  | 57,058,513 |
| SNP_320 | GGTGCTATTCCCGTTACCTTAC | GATGCGTTCCAGGTCTTTCA | SNP |  | 57,063,444 |
| CAPS_9 | TTCGGTGAAGCTGGAGAAAC | GACGTGACCCAAACCAATCT | CAPS | HaeIII | 57,073,201 |

**S1 Table. Primers for fine mapping.**
