# Supplementary material for: Identification of Dw1, a Regulator of Sorghum Stem Internode Length
Source: PLoS One. 2016 Mar 10;11(3):e0151271. doi: 10.1371/journal.pone.0151271 (PMC4786228; doi:10.1371/journal.pone.0151271)
Supplement: S2 Table — (DOCX) [file pone.0151271.s006.docx]

**S2 Table. Primers for Sequencing of Candidate Genes**

| **Gene** | **Primer** | **Primer Sequence** |
| --- | --- | --- |
| Sobic.009G229500 | **240_AmpFor3** | CTTGGCGCTAGTTCCTACTT |
|  | **240_AmpRev4** | GCAGTTGGAGGAGCTAAGAAA |
|  | 240_AmpFor2 | CTGCTGAGCTGAGTATGGATATG |
|  | SNP_240B Rev | GGGAGAAGGCCGTGATATAAA |
|  | 240_AmpRev3 | TGGAACTGTGGAAGGCAATAG |
| Sobic.009G229600 | **250_AmpFor5** | AGCTGACCTGGCAATACTTAC |
|  | **250_SeqRev6** | GGCAGACTCTCTAAGCTGATTT |
|  | SNP_250D For | GAGCTGAAGAGCTTCCCTTG |
|  | SNP_250F Rev | CTGTCGAAGTTCTTCTCGATGT |
|  | SNP_250D Rev | CTCTTAGCCAGCACTAGCAATC |
|  | 250_SeqFor8 | AATCCTCTGTCCTGCCATTC |
|  | **SNP_250 For** | GCGGACATCCAACTCTGATAG |
|  | **250_SeqRev1** | CACATTGCATCACCAACATCAA |
|  | SNP_250 Rev | AGTCTTCAGGTTGCTCCATTAC |
|  | SNP_250G For | CCATAACTGCAGTGCATGATTC |
|  | SNP_250G Rev | TATGTGCCTCACCTTCCTTTC |
|  | 250_SeqFor4 | TCAGTAGCCCACAGGAGAATAG |
|  | 250_SeqFor5 | GATCGCCTAACAGCATGTAATTC |
|  | 250_SeqRev7 | CAAATGGCACCAGGACCTATTA |
|  | 250_SeqRev8 | TACCCTACGCATGAGGATAAGA |
|  | **250_SeqFor1** | GCGGCTAGTGTTGAGGATTTA |
|  | **SNP_250C Rev** | CTGTTTAGCCCGTCCTTCTT |
|  | 250_SeqFor6 | GCTTTACTCCAGATGCACAAATAG |
|  | 250_SeqRev9 | GAAGTTCACTGGCCTGAAGTATTA |
|  | 250_SeqFor9 | CGCCCTGAACAAGATGTTATG |
|  | 250_SeqRev11 | CCAATGCCTCAGCCTCTTTA |
|  | **SNP_250C For** | GGTTGTTTCAGGCTGCTTTC |
|  | **250_SeqRev3** | TGGCAGAGTTCACCCAAATAA |
|  | SNP_250E For | CTAAAGTTCCACTTCCCGATCA |
|  | SNP_250E Rev | CACAGGTTGGCAGCAGATA |
|  | 250_SeqFor7 | TGCAGGAAGCAGAAGCTAAA |
|  | 250_SeqRev10 | GGAGTCATGGTCCTCAGATAATAC |
|  | 250_AmpFor3 | CCTATACCTCCCACGTTCAAATAC |
|  | 250_AmpFor4 | GCTGTCTAGTTCTGGCAGTATAA |
|  | **250_SeqFor3** | GTCTTCCTGGCTAACTTCTACTG |
|  | **250_AmpRev4** | CAAGAATGGAACTGGCAACATAC |
|  | SNP_250B For | GCTGAATTGGAAGCTCTGAAAC |
|  | 250_SeqFor10 | GCAGTTGGAGGAGCTAAGAAA |
| Sobic.009G229700 | **SNP_260C For** | CACGATACGATTCCACCGAATTA |
|  | **SNP_260C Rev** | CAACCAAGCAGTTAGGCTCATA |
| Sobic.009G229800 | **270_AmpFor3** | GCACGTACGTACAATCAAGTTATG |
|  | **270_AmpRev1** | CACAGCCTACATCATCAGTAAGA |
|  | 270_AmpFor2 | GAGCAACCGTGTGTGTTTAC |
|  | 270_SeqRev1 | GTAAAGATGCCCAGTTTCAAGTC |
|  | SNP_270 For | GATATGTGGACGACAGGATCAG |
|  | SNP_270B For | GGACTAACACACGCTTCTCTAC |
|  | SNP_270 Rev | ATTGAGCAGTCGAAGGAAGG |
|  | 270_SeqFor1 | CAGGCATCCTACCCACTTTAC |
|  | SNP_270B Rev | CATCTTGCTTCTCCCTGGATAC |
|  | 270_SeqFor2 | ACCAACTCTCCATTGATTCTCC |
|  | 270_SeqRev3 | CCAGCTGCAAATAGCCAAATAG |
|  | 270_SeqRev4 | GCCCATCTACTTTGCTGTTTAG |
|  | 270_SeqRev5 | GGAACCTCTTGCTCAGGTATAG |
|  | 270_SeqFor2 | CGATACACTCCCACCCATTT |
|  | 270_AmpFor4 | CTCTCACTCAGCTCTCTCTTTC |
|  | 270_SeqRev2 | CCTGCCATTTGAGAACAGAAAC |
| Sobic.009G229900 | **280_AmpFor1** | CGTGCTCAGTGCTCTTTATATTTG |
|  | **SNP_280B Rev** | CAGGAACCTCCATTTCCATGA |
|  | 280_SeqRev4 | CGCCTGAACGAGAACCTTT |
|  | SNP_280B For | CTGTCCAACGCCATCACTAA |
|  | 280_SeqRev3 | CAGTGGTGTTTAACGCTGTATTG |
|  | **SNP_280 For** | CCTTGACAGTTTCGAGGGTAAG |
|  | **280_AmpRev2** | CAGCAAGGGTAGCATTAGAAGAG |
|  | 280_SeqFor3 | CCCAGTCGTCCCTAGACATAA |
|  | SNP_280 Rev | CTGCGTTCTTGGGATCTTGT |
|  | 280_SeqFor4 | GGCCTGTGGGATTTGTACTT |
| Sobic.009G230000 | **290_AmpFor4** | CCAGCATCGTCAACGTAACT |
|  | **290_AmpRev1** | CTCTTAATCGGTGGATGAGTACAA |
|  | 290_AmpFor1 | CTCGTGAACCGACGATTTCT |
|  | 290_SeqRev2 | GTGGGCGGTGGGATTTATAG |
|  | SNP_290 For | TGGTCCACCTGCTCTACA |
|  | 290_SeqRev1 | CGGGCTCCAGTATCTCCA |
|  | 290_SeqFor1 | TTGGCTCGTCCCATGATTT |
| Sobic.009G230100 | **300_AmpFor2** | CTGGACTAGTTTCTGGTTCGTTAC |
|  | **SNP_300B Rev** | CCTGAAGCAGGTCTCTGAATG |
|  | 300_SeqRev3 | GCAGGGTAGATTGAGAGCTTAC |
|  | 300_SeqRev 4 | CCATGTAGAGCCACCTCATAGA |
|  | **SNP_300B For** | TCTGATGCGACCGATCTTTG |
|  | **300_AmpRev1** | CCAATGGGTTTACCGTCTACTG |
|  | 300_SeqFor3 | GACACCCTGTCGCGAATAAA |
|  | SNP_300 For | CCTGAGTTGTTCCTGCAGATAG |
|  | SNP_300 Rev | GGCGCGTGTCATTAGTAGAA |
|  | 300_SeqFor4 | CGAGATCTATGAGGTGGCTCTA |
|  | 300_SeqRev5 | GCAGTATGCTAGTCCCATGATAA |
|  | 300_SeqFor5 | CCAGCATCGTCAACGTAACT |
|  | 300_SeqRev6 | GATCCAGCAAGGAGGCTATAC |
