# Supplementary material for: Identification of Dw1, a Regulator of Sorghum Stem Internode Length
Source: PLoS One. 2016 Mar 10;11(3):e0151271. doi: 10.1371/journal.pone.0151271 (PMC4786228; doi:10.1371/journal.pone.0151271)
Supplement: S3 Table — (DOCX) [file pone.0151271.s007.docx]

| **Primer name** | **Primer sequence** |
| --- | --- |
| **270_AmpFor4** | CTCTCACTCAGCTCTCTCTTTC |
| **270_AmpRev2** | ACGATTGGAGTGTCTACAAAGAG |
| SNP_270 Rev | ATTGAGCAGTCGAAGGAAGG |
| 270_SeqFor1 | CAGGCATCCTACCCACTTTAC |
| SNP_270B Rev | CATCTTGCTTCTCCCTGGATAC |
| 270_SeqFor2 | ACCAACTCTCCATTGATTCTCC |
| qRT_4R | CAAGAATGGCCAGGAAGAGAT |
| qRT_9R | CCCAACTGAAGACATCTCTGAC |
| qRT_20F | GCGGTCCAACGTCTAATATGT |
| qRT_10F | GCAGGACAGGCAAAGTAGAT |
| qRT_21R | CATCTTGCTTCTCCCTGGATAC |
| qRT_29F | GCGGTCCAACGTCTAATATGT |
| qRT_28R | TGGTCTCTTGCTCAGGAATTG |

**S3 Table. Primers for Amplifying cDNA of Sobic.009G229800**
