# Supplementary material for: Identification of Dw1, a Regulator of Sorghum Stem Internode Length
Source: PLoS One. 2016 Mar 10;11(3):e0151271. doi: 10.1371/journal.pone.0151271 (PMC4786228; doi:10.1371/journal.pone.0151271)
Supplement: S4 Table — (DOCX) [file pone.0151271.s008.docx]

**S4 Table. *Dw1* QTL for Each Trait for Hegari x 80M F_2_**

| **Trait** | **# of QTL** | **QTL at Dw1?** | **Peak (Mbp)** | **Peak LOD** | **Additive** | **Dominance** | **R2** |
| --- | --- | --- | --- | --- | --- | --- | --- |
| Average Internode Length | 4 | Yes | 56.64 | 21.8 | -27.3763 | 6.4375 | 0.2186 |
| Length of Internode 5 | 4 | Yes | 56.64 | 7.67 | -18.1324 | 8.5803 | 0.1144 |
| Length of Internode 7 | 4 | Yes | 56.47 | 16.05 | -29.4216 | 10.1362 | 0.2158 |
| Length of Internode 10 | 4 | Yes | 57.07 | 13.09 | -29.6994 | 11.5228 | 0.2388 |
| Total Length | 4 | Yes | 57.07 | 18.91 | -46.3611 | 23.7702 | 0.3695 |
| Stem Fresh Weight | 3 | Yes | 57.07 | 9.91 | -69.9536 | 23.7784 | 0.1869 |
| Stem Dry Weight | 2 | Yes | 57.07 | 8.04 | -15.8367 | 7.1609 | 0.1614 |
| Diameter | 2 | No |  |  |  |  |  |
| Stem Fresh Weight/Stem Length | 3 | No |  |  |  |  |  |
