# Supplementary material for: Identification of Dw1, a Regulator of Sorghum Stem Internode Length
Source: PLoS One. 2016 Mar 10;11(3):e0151271. doi: 10.1371/journal.pone.0151271 (PMC4786228; doi:10.1371/journal.pone.0151271)
Supplement: S5 Table — (DOCX) [file pone.0151271.s009.docx]

S5 Table. QTL for Average Internode Length Identified using MQM in R/qtl

| **QTL** | **Chr** | **Peak (cM)** | **LOD** | **Peak (Mbp)** | **Additive** | **Dominance** | **% Variation** | ***Dw* locus** |
| --- | --- | --- | --- | --- | --- | --- | --- | --- |
| 1 | 1 | 97.1 | 7.31 | 54.67 | -12.66 | -3.956 | 3.693 | *Dw01_54.7* |
| 2 | 6 | 41.1 | 20.274 | 42.64 | 23.531 | 2.016 | 11.849 | *Dw2* |
| 3 | 7 | 58.8 | 50.968 | 55.15 | -39.248 | 22.749 | 43.127 | *Dw07_55.1* |
| 4 | 9 | 107.2 | 31.628 | 57.07 | 26.329 | 7.254 | 21.11 | *Dw1* |
| 5 | 10 | 19.3 | 4.883 | 3.17 | 8.839 | -8.392 | 2.403 | *Dw10_3.2* |

| **QTL** | **LOD** | **% Variation** | **Add:Add** | **Add:Dom** | **Dom:Add** | **Dom:Dom** | ***Dw* locus** |
| --- | --- | --- | --- | --- | --- | --- | --- |
| 3:4 | 5.593 | 2.773 | 12.773 | 5.845 | -11.038 | -4.486 | *Dw07_55.1*:*Dw1* |
