# Supplementary material for: Identification of Dw1, a Regulator of Sorghum Stem Internode Length
Source: PLoS One. 2016 Mar 10;11(3):e0151271. doi: 10.1371/journal.pone.0151271 (PMC4786228; doi:10.1371/journal.pone.0151271)
Supplement: S6 Table — (DOCX) [file pone.0151271.s010.docx]

**S6 Table. Maize and Rice Homologs of the Seven Genes in the Delimited *Dw1* Region**

| **Sorghum** | **Maize** | **Rice** |
| --- | --- | --- |
| Sobic.009G229500 | GRMZM2G405706 | LOC_Os05g48610 |
| Sobic.009G229600 | N/A | LOC_Os05g48620 |
| Sobic.009G229700 | GRMZM2G451224 | LOC_Os05g48630 |
| Sobic.009G229800 | GRMZM2G079832; GRMZM2G060467 | LOC_Os01g01390 |
| Sobic.009G229900 | GRMZM2G359664; GRMZM2G377615 | LOC_Os05g48640 |
| Sobic.009G230000 | GRMZM2G377613 | LOC_Os05g48650 |
| Sobic.009G230100 | GRMZM2G079583 | LOC_Os05g48660 |
