# Supplementary material for: Identification of Dw1, a Regulator of Sorghum Stem Internode Length
Source: PLoS One. 2016 Mar 10;11(3):e0151271. doi: 10.1371/journal.pone.0151271 (PMC4786228; doi:10.1371/journal.pone.0151271)
Supplement: S10 Table — (DOCX) [file pone.0151271.s014.docx]

**S9 Table. Summary of Protein Function Searches**

| **Program** | **Program Description** | **Annotations** |
| --- | --- | --- |
| BLAST-Arabidopsis homolog (TAIR) | finds homologs of subject | involved in: biological _process;  located in: nucleus (predicted), plasma membrane (experimental);  closest paralog: AT5G52430 hydroxyproline-rich glycoprotein |
| NCBI-conserved domain | database search of domains and proteins | large tegument protein |
| PSIPRED-MEMSAT-SVM | membrane helix prediction | pore-lining/transmembrane residues 263-278 |
| PSIPRED-DISOPRED | predicts disorder based on homologs | highly disordered; possibly protein binding |
| PONDR | predicts disordered regions | ~52% disordered; two long regions of disorder |
| FoldIndex | predicts disordered regions | ~44% disordered |
